# Atypical CD5 and CD10 coexpression in a splenic marginal zone lymphoma

**DOI:** 10.1002/jha2.155

**Published:** 2021-02-06

**Authors:** Santiago Gimenez De Mestral, François Delhommeau, Bettina Fabiani, Pascale Cervera, Ludovic Suner

**Affiliations:** ^1^ Pathology Department AP‐HP Hôpital Saint‐Antoine Faculté de Médecine Sorbonne Université Paris France; ^2^ Sorbonne Université Inserm Centre de Recherche Saint‐Antoine CRSA AP‐HP Hôpital Saint‐Antoine Hématologie Biologique Paris France

A 73‐year‐old man came to our institution for tiredness, anorexia, and weight loss. Complete blood count showed mild anemia and leukocytosis with normal platelets (hemoglobin = 106 g/L, leukocytes = 10.7 × 10^9^/L, platelets 229 × 10^9^/L). Other laboratory tests indicated particularly increased lactate dehydrogenase (1314 UI/L) and C‐reactive protein (167 mg/L). A CT‐scan revealed multiple abdominal and pelvic adenopathies together with a splenomegaly. Flow cytometric analysis completed on a bone marrow aspirate and on a blood sample was inconclusive. A splenectomy was then performed.


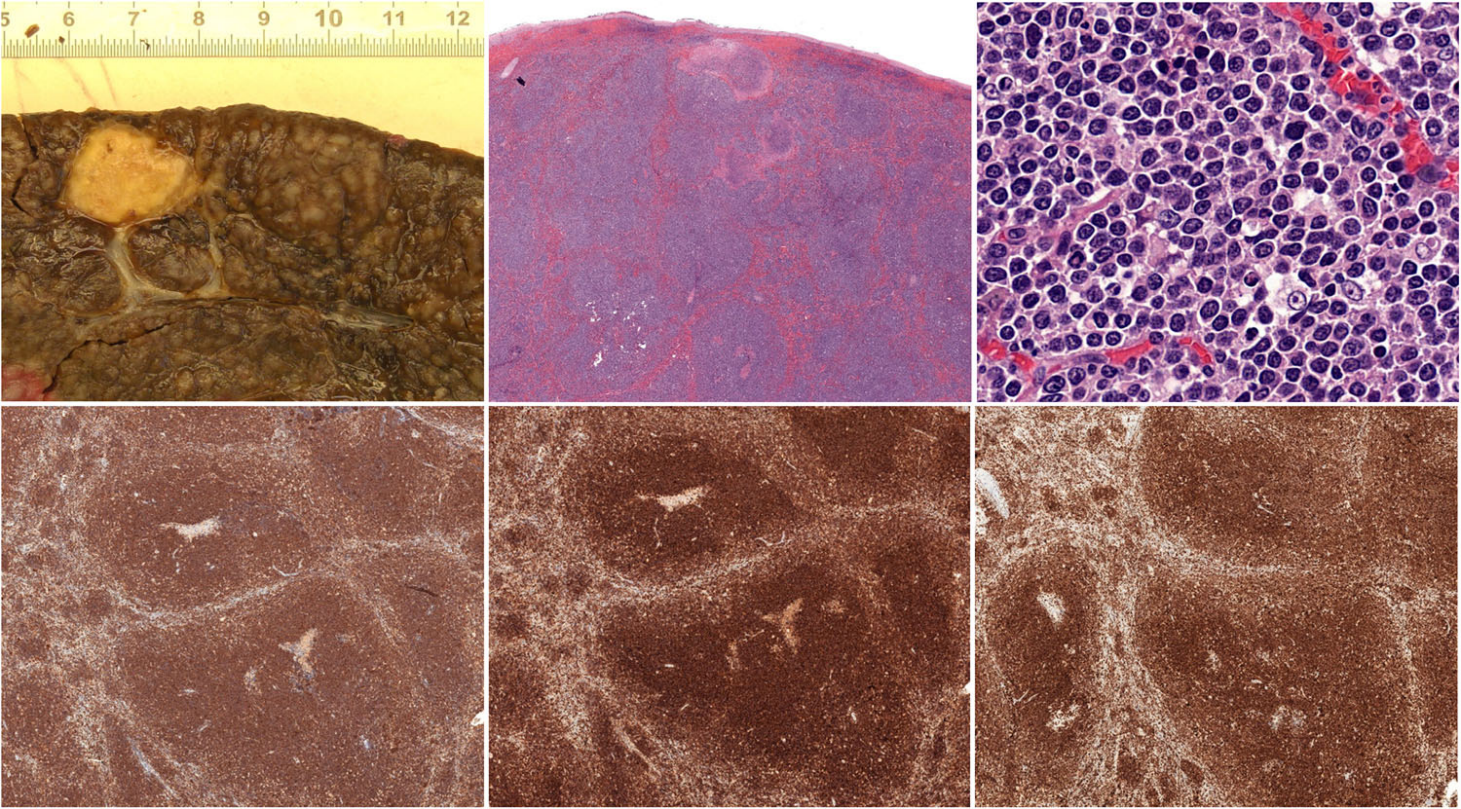


The spleen weighed 2800 g and measured 29 cm, with diffuse hyperplasia of the white pulp appearing as pale nodules with necrotic sectors and large infiltrated lymph nodes in the hilum (top left). Histopathological analysis showed a lymphoid proliferation with nodular architecture and necrotic foci (top middle, 150x). Cells were medium‐sized with a discreetly irregular nucleus (top right, 400x), and a CD20+ (bottom left, 150x), CD5+ (bottom middle, 150x), CD3−, BCL2+, CD10+ (bottom right, 150x), CD23−, Cyclin D1−, SOX11−, BCL6−, and HGAL− phenotype. The Ki67 proliferation index was about 50%, and a hyperplastic and dissociated network of CD21+ dendritic follicular cells was present in most nodules. FISH revealed an amplification of the centrometric part of 18q in 18q21.33. We finally completed the diagnosis with a targeted sequencing analysis of 44 genes frequently mutated in lymphoid malignancies. A *TP53* mutation (I255F; VAF = 52%) was detected alongside a discrete *NOTCH2* mutation (R234C; VAF = 5%), suggesting a poor prognosis.

CD5 and CD10 coexpression is very uncommon in B‐cell lymphomas, and these entities may represent a challenging diagnosis. Cases occurring in diverse low‐grade B‐cell lymphoma subtypes but also in aggressive lymphomas, such as large B‐cell lymphoma and B‐cell lymphoblastic leukemia/lymphoma have been reported. In our case, the elements taken together suggest a transformed small B‐cell lymphoma. CLL and MCL are excluded by histopathological exams. Despite CD10 positivity, a strong maker of germinal center origin, molecular and genetic analyses are not in favor of a follicular lymphoma. Instead, combined mutation of *NOTCH2* (upstream the PEST domain) and *TP53* supports the hypothesis of a lymphoma evolving from a SMZL.

Patient treatment comprised six cycles of R‐CHOP and four therapeutic lumbar punctures with methotrexate and methylprednisolone. Three months after achieving complete remission, his condition is good, and his last PET‐CT evaluation shows no sign of residual metabolically active tissue.

## AUTHOR CONTRIBUTIONS

Santiago Gimenez De Mestral performed histopathological analysis and collected the images. François Delhommeau performed cytological analysis. Bettina Fabiani performed histopathological analysis. Pascale Cervera performed FISH and molecular analysis. Ludovic Suner collected clinical data, collected images, and corrected the manuscript. All the authors participated in the writing of the manuscript.

## Data Availability

The data that support the findings of this study are available from the corresponding author upon reasonable request.

